# 

**Published:** 1897-11

**Authors:** 


### CONTENTS.

ORIGINAL COMMUNICATIONS. PAGE
Diphtheria. By GARDNER T. SWARTS, M.D. . . . . . . . . . . . . . . . . . . 705
Relations of Chemistry to Dentistry. By J. S. CASSIDY, D.D.S., Covington, Ky. 719
"The Mouth is the *Via Natura* for the Entrance of Disease." By M. V. BALL, M.D. . . . . . . . . . . . . . . . . . . . . . . . . . . . . . . 724
Examining Boards: an Open Letter. By B. F. ARRINGTON, Goldsboro, N.C. . . 729

ABSTRACTS AND TRANSLATIONS.
Formaldehyde. By H. C. WOOD, M.D., LL.D., Philadelphia, Pa. . . . . . . . 734

REPORTS OF SOCIETY MEETINGS.
American Dental Association . . . . . . . . . . . . . . . . . . . . . . 740
American Academy of Dental Science . . . . . . . . . . . . . . . . . . . 754
Odontological Society of Pennsylvania . . . . . . . . . . . . . . . . . . 761

EDITORIAL.
The Reign of Law . . . . . . . . . . . . . . . . . . . . . . . . . . . 768

BIBLIOGRAPHY . . . . . . . . . . . . . . . . . . . . . . . . . . . . . 772

OBITUARY.
Dr. Francis Peabody . . . . . . . . . . . . . . . . . . . . . . . . . . 775
Professor Frank Abbott . . . . . . . . . . . . . . . . . . . . . . . . 775
Resolutions of Respect to Dr. E. Magitot . . . . . . . . . . . . . . . . 776

